# Grape seed powder increases gastrointestinal motility

**DOI:** 10.7150/ijms.72529

**Published:** 2022-05-21

**Authors:** Na Ri Choi, Jeong Nam Kim, Min Ji Kwon, Jong Rok Lee, Sang Chan Kim, Min Jae Lee, Woo-gyun Choi, Byung Joo Kim

**Affiliations:** 1 Division of Longevity and Biofunctional Medicine, Pusan National University School of Korean Medicine, Yangsan 50612, Republic of Korea.; 2 Department of Pharmaceutical Engineering, Daegu Haany University, Gyeongsan 38610, Republic of Korea.; 3 College of Oriental Medicine, Daegu Haany University, Gyeongsan 38610, Republic of Korea.; 4 College of Veterinary Medicine, Kangwon National University, Chuncheon 24341, Republic of Korea.

**Keywords:** Grape seed, Bioactive product, Interstitial cell of Cajal, Gastrointestinal motility, ICC

## Abstract

Grape seed is an important natural bioactive product with various health benefits. Interstitial cells of Cajal (ICCs) are pacemaker cells in the gastrointestinal (GI) tract. The present study investigated the effects of grape seed powder (GSP) on ICC properties and GI motility. GSP depolarized the pacemaker potentials of ICCs in a dose‑dependent manner. Y25130 or SB269970 slightly inhibited GSP‑induced effects. However, Y25130 and SB269970 together completely blocked GSP-induced effects. In the presence of inhibitors of protein kinase C, protein kinase A, or mitogen-activated protein kinase, GSP‑induced ICC depolarization was inhibited. GSP increased the intestinal transit rate in normal mice and in mice with acetic acid-induced GI motility disorder. In addition, the levels of motilin and substance P were elevated after GSP dosing. These results demonstrate that GSP can regulate GI motility, and therefore, it is a potential therapeutic agent for treating GI motility disorders.

## Introduction

Bioactive substances isolated from food ingredients may be used as natural alternative medicine for treating various diseases 1. Grape seed, a major source of catechins and procyanidins, is readily available, inexpensive, and beneficial to human health 1,2. Grape seed powder (GSP) has antioxidant, anti-inflammatory, antidiabetic, anti-obesity, anticancer, anti-aging, and antimicrobial properties 3-7. Therefore, it has a potential role as a substitute or complement to drugs against different diseases.

Most mammals obtain energy from food nutrients through the gastrointestinal (GI) tract 8. Therefore, food intake, digestion, motility, secretion, and absorption are key physiological processes contributing to the maintenance of energy homeostasis 9. GI motility changes with food and is regulated by smooth muscle, extrinsic and intrinsic neurons, interstitial cells of Cajal (ICCs), and several hormones 10,11. ICCs are the pacemakers of the GI system, and they induce pacemaker potentials spontaneously 11,12. Therefore, ICCs are key regulators of GI motility. However, little is known about the effects of GSP on ICC properties and GI motility. Therefore, in the present study, we investigated the regulatory effects of GSP on ICC properties and GI motility.

## Materials and Methods

### Materials, Instruments and Reagents

The grape seed powder (GSP) used in this study was a commercial product made from Govinda Natur GmbH (Neuhofen, Germany). ACQUITY^Tm^ ultra-performance liquid chromatography (UPLC) system (USA), ACQUITY^TM^ photodiode array detector (PDA), ACQUITY^TM^ BEH C_18_ column (1.7 µm, 2.1 mm × 100 mm), and Empower chromatography data system were purchased from Waters (Milford, MA, USA). In addition, the sample extractor was ultrasonic cleaner 8210R-DHT from Branson Company. The reagents used for HPLC analysis were methanol from Junsei, acetonitrile from JT-BAKER, water (Tertiary distilled water), and DMSO. The standards were from Cayman Chemical Company (Ann Arbor, MI, USA) and Sigma-Aldrich (St. Louis, MO, USA).

### Preparation of standard solutions

The required amounts of (-) epicatechin, (-) epicatechin gallate, resveratrol, and nomilin were measured accurately and dissolved in DMSO and methanol to prepare 1 mg/ml standard solutions. The standard solutions were successively diluted with methanol to prepare 12.5, 25, 50, and 100 μg/ml solutions. The determination coefficient (R_2_) values of all the standards materials were > 0.999.

### Preparation of the test liquid for quantitative analysis

The test liquid was prepared by soaking 1 g of grape seed powder in 10 ml methanol, followed by microwave extraction for 1 h. The powder was filtered using a membrane filter of < 0.2 μm diameter. The filtrate was used as the test liquid.

### Quantitation of GSP

UPLC analysis was carried out at room temperature. (-) Epicatechin/(-) epicatechin gallate, resveratrol, and nomilin were measured at 280 nm, 320 nm, and 254 nm respectively. The mobile phase was a mixture of water and acetonitrile containing 0.1 % formic acid. The sample (2 μl) was injected at a flow rate of 0.4 ml/min. Qualitative and quantitative analyses were carried out using retention time and peak area, respectively. The analysis conditions are presented in Table 1.

### Animals

ICR mice were purchased from the Samtako Bio (Osan, Republic of Korea). Males and females (3‑7 days old) were used for the ICC experiment and only males (8-week old) were used for the ITR experiment as well as for the intestinal hormone and protein experiments. All mice were housed in a specific pathogen‑free laboratory environment under a controlled temperature (21-23 °C) and humidity (50-60 %) with day and night cycles and ad libitum access to normal diet and autoclaved water. The Institutional Animal Care and Use Committee at Pusan National University (approval no. PNU‑2020‑2831) approved all animal care and experiments (Busan, Republic of Korea). In addition, all the animals were treated in accordance with the Guide for the Care and Use of Laboratory Animals.

### Drugs

Y25130, RS39604, SB269970, Go6976, SQ22536, PD98059, SB203580, and JNK II inhibitor were purchased from Tocris Bioscience (Bristol, UK), and all the other drugs were obtained from Sigma‑Aldrich (St. Louis, MO, USA). We used distilled water to dissolve GSP in experiments. A stock(X10) was prepared and used after dilution to a predetermined concentration.

### Preparation of ICCs

The small intestinal mucosae were removed and small tissue strips of intestinal muscle were equilibrated for 30 min in Ca^2+^‑free Hank's solution. Next, the cells were dispersed in an enzyme solution and cultured in a smooth muscle growth medium (Lonza, Basel, Switzerland) at 37 °C in a 95 % O_2_ incubator. Finally, the ICCs were identified 12 by using the patch‑clamp technique, which showed the network‑like structures in culture.

### Electrophysiological experiments

We used the whole‑cell electrophysiological method to investigate the pacemaker potentials of ICCs. The bath solution contained 5 mM KCl, 135 mM NaCl, 2 mM CaCl_2_, 10 mM glucose, 1.2 mM MgCl_2_, and 10 mM HEPES (pH 7.4), and the pipette solution contained 140 mM KCl, 5 mM MgCl_2_, 2.7 mM K_2_ATP, 0.1 mM NaGTP, 2.5 mM creatine phosphate disodium, 5 mM HEPES, and 0.1 mM EGTA (pH 7.2). Electrophysiological experiments were conducted with Axopatch I‑D and 200B amplifiers (Axon Instruments, San Jose, CA, USA). All experiments were performed at 30-31 °C.

### ITR measurement

Evans blue (5 %, w/v; 0.1 ml/kg) was intragastrically administered 30 min after the intragastric administration of GSP to ICR mice. ITR was measured 30 min after the administration of Evans blue.

### GI Motility Dysfunction (GMD) model

GMD mice models were established by treating mice with acetic acid (AA) (0.5 % in saline, w/v) to induce peritoneal irritation. After the AA treatment, the mice were stabilized in a cage for 30 min, and then GSP was administered to measure ITR 13,14.

### Measurement of serum intestinal hormone levels

After feeding the mice GSP (0.5 g/kg) once a day for 5 days, the serum levels of intestinal hormones, such as motilin (MTL), substance P (SP), somatostatin (SS), and vasoactive intestinal polypeptide (VIP) were measured by radioimmunoassay (Abbkine, Wuhan, China).

### Western blotting

After feeding the mice GSP (0.5 g/kg) once a day for 5 days, the small intestine samples were collected and prepared by incubating in RIPA buffer. The total protein extracted and an equal amount of protein from the samples were separated using SDS‑PAGE and then transferred to polyvinylidene difluoride membranes. The membranes were probed with the indicated antibodies. Anti‑transmembrane protein 16A (TMEM16A; Abcam, Cambridge, UK), anti‑c‑kit (Cell Signaling Technology, Denver, MA, USA), anti‑transient receptor potential melastatin 7 (TRPM7; Abcam, Cambridge, UK), and anti‑β‑actin (Santa Cruz Biotechnology, Dallas, TX) antibodies were used. All other procedures were conducted as previously described 12.

### Statistical analysis

Data are expressed as mean ± standard error of mean. For multiple comparison analysis, we used one‑way analysis of variance (ANOVA) with Turkey post hoc comparison and when only two groups were compared, Student's t‑test for paired data was used. A *p* value < 0.05 indicated statistical significance.

## Results

### Functional constituents of GSP

**Figure 1:**
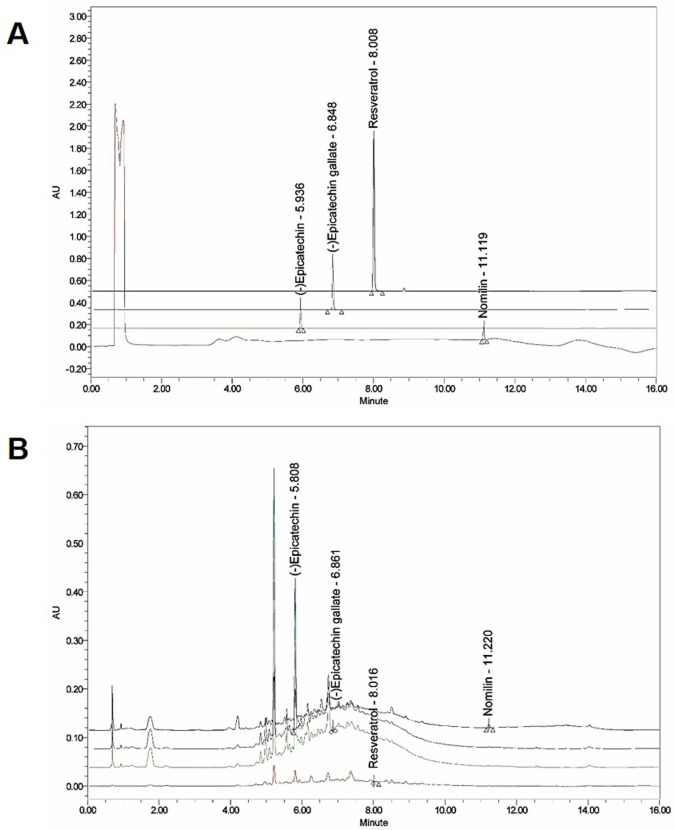
Ultra‑performance liquid chromatography (UPLC) chromatogram of the composition of grape seed powder. (A) UPLC profile of the commercial standard compounds. (B) UPLC profile of the four major compounds in GSP. Chromatograms were obtained at 280 nm ((-) epicatechin and (-) epicatechin gallate), 320 nm (resveratrol), and 254 nm (nomilin).

The presence of (-) epicatechin, (-) epicatechin gallate, resveratrol, and nomilin in GSP was established by HPLC and their levels were quantified using calibration curves obtained from standards (Table 2; Fig. 1). The validation of the method used confirmed its reliability and stability.

### Effects of GSP on ICC pacemaker potentials

**Figure 2:**
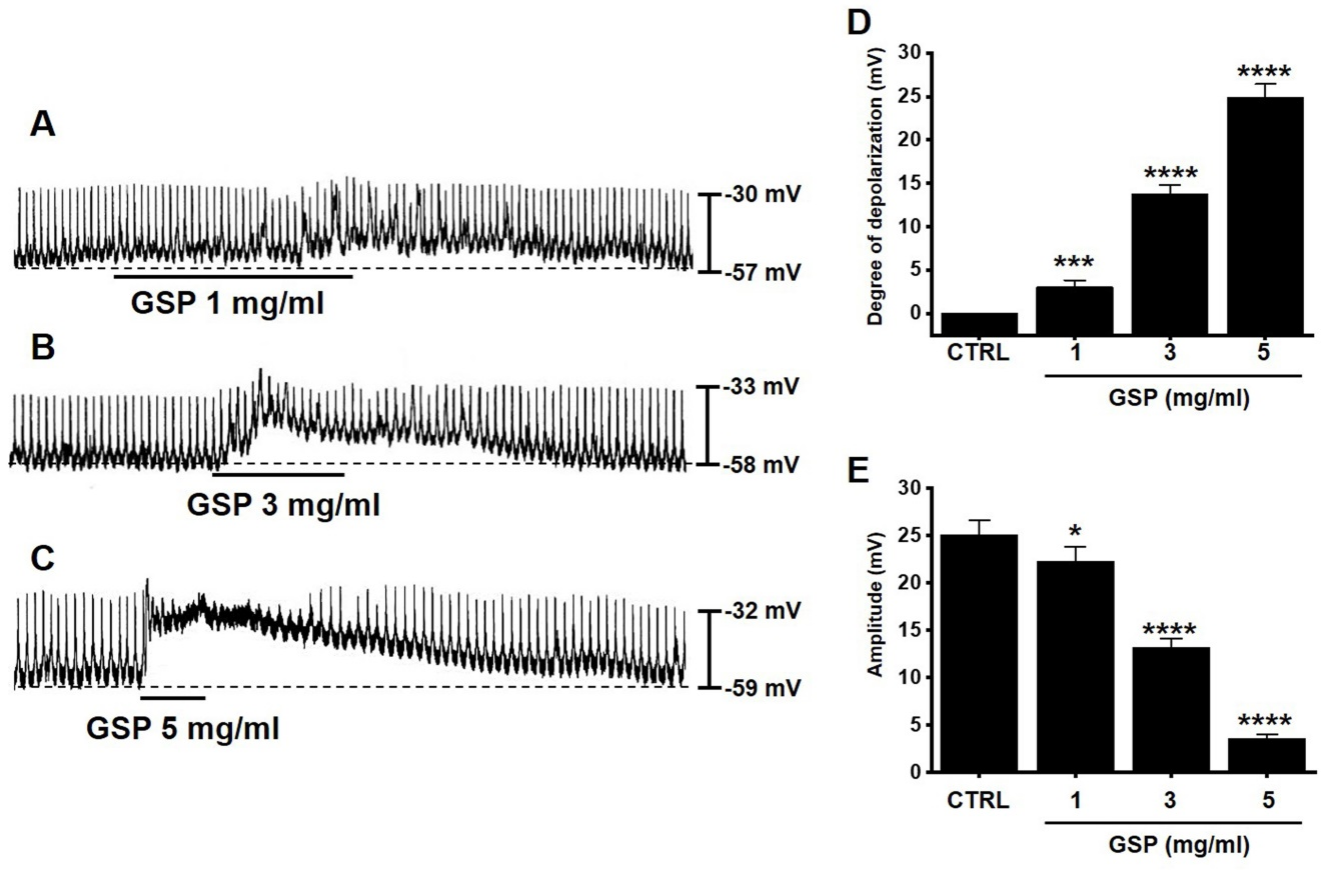
Effects of GSP on the pacemaker potentials of interstitial cells of Cajal. (A‑C) GSP depolarized pacemaker potentials and suppressed the amplitude. (D and E) Summary of responses to GSP. Mean ± standard error of mean. ***p < 0.001. ****p < 0.0001. CTRL, control; GSP, Grape seed powder.

ICC generated pacemaker potentials with a resting membrane potential of ‑57.3 ± 1.2 mV and amplitude of 25.1 ± 1.6 mV under the current clamp mode (I = 0) (Fig. 2). GSP (1-5 mg/ml) depolarized pacemaker potentials and decreased the amplitudes in a concentration‑dependent manner (Fig. 2A‑2C). The degrees of depolarization by GSP were 3.0 ± 0.8 mV (*p* < 0.001) at 1 mg/ml, 13.8 ± 1.0 mV (*p* < 0.0001) at 3 mg/ml, and 24.9 ± 1.5 mV (*p* < 0.0001) at 5 mg/ml (Fig. 2D), and the amplitudes were 22.2 ± 1.6 mV (*p* < 0.05) at 1 mg/ml, 13.2 ± 1.0 mV (*P* < 0.0001) at 3 mg/ml, and 3.6 ± 0.5 mV (*p* < 0.0001) at 5 mg/ml (Fig. 2E). These results showed that GSP dose‑dependently depolarized ICC pacemaker potentials.

### Effects of 5-HT antagonists on GSP‑induced ICC depolarization

**Figure 3:**
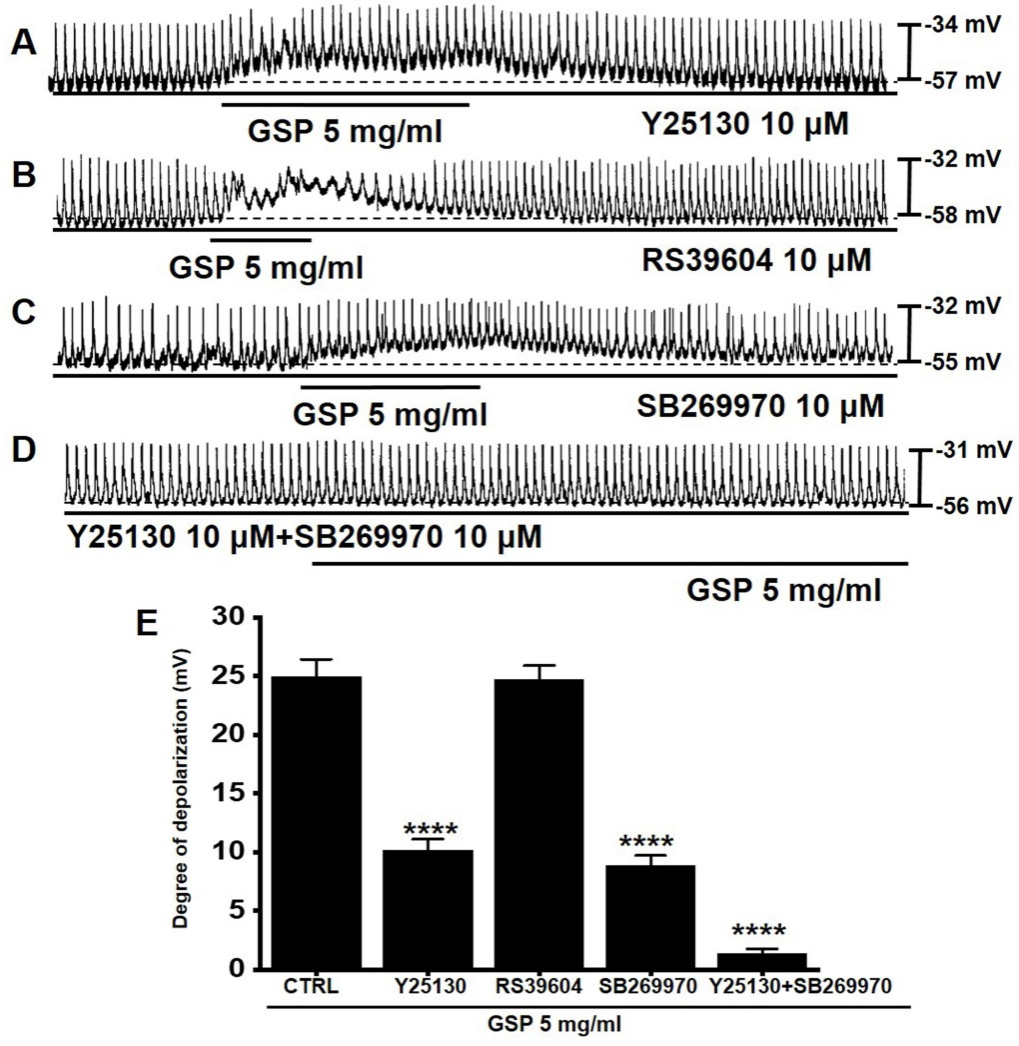
Effects of 5-HT receptor antagonists on GSP‑induced depolarization. (A and C) Y25130 or SB269970 blocked GSP‑induced depolarization. (B) RS39604 did not inhibit GSP‑induced depolarization. (D) Y25130 and SB269970 together completely blocked SM‑induced depolarization. (E) Summary of responses to GSP. Mean ± SEM. ****p < 0.0001. CTRL, control; GSP, Grape seed powder.

5-HT can influence several GI functions, particularly motility control 15,16. Previous studies have shown that there are three 5-HT receptors (3, 4, and 7) in the small intestinal ICC 17,18. To investigate if the serotonergic pathway was involved in GSP‑induced ICC depolarization, antagonists to 5-HT_3_ (Y25130), 5-HT_4_ (RS39604), 5-HT_7_ (SB269970), or the mixture was administered to ICCs (Fig. 3). Pretreatment with Y25130 or SB269970 slightly inhibited the GSP‑induced effects (Fig. 3A and 3C). However, pretreatment with RS39604 had no effect on GSP‑induced effects (Fig. 3B). In addition, pretreatment with Y25130 and SB269970 together completely blocked GSP-induced effects (Fig. 3D). The degrees of depolarization by GSP were 10.2 ± 0.9 mV (p < 0.0001) with Y25130, 24.7 ± 1.2 mV with RS39604, 8.8 ± 0.9 mV (*p* < 0.0001) with SB269970, and 1.4 ± 0.4 mV (p < 0.0001) with both Y25130 and SB269970 (Fig. 3E). The results showed that GSP influenced ICC pacemaker potentials through both 5-HT_3_ and _7_ receptors.

### PKC or PKA pathway antagonists influence GSP‑induced ICC depolarization

**Figure 4:**
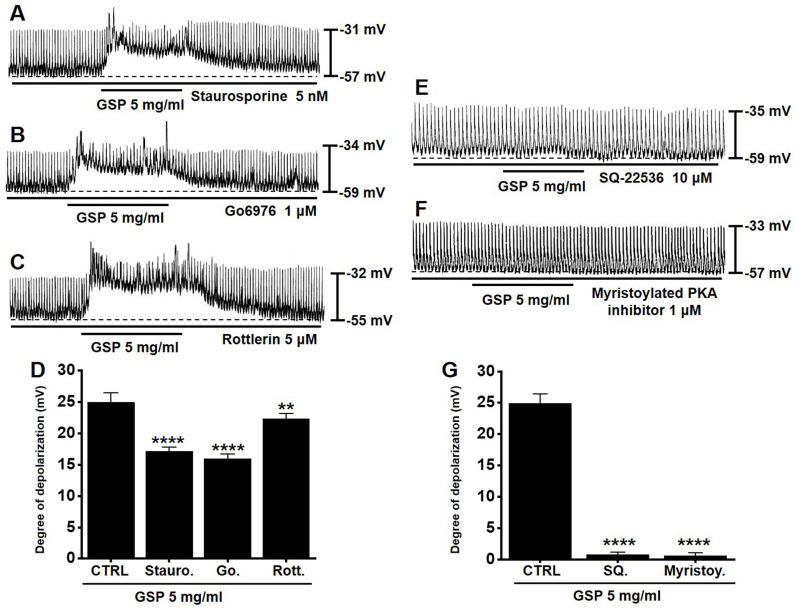
Effects of the inhibition of protein kinase C and protein kinase A on GSP‑induced depolarization. (A-C) PKC inhibitors (staurosporine, Go6976, and rottlerin) slightly inhibited GSP‑induced depolarization. (E and F) PKA inhibitors (SQ22536 and myristoylated) completely inhibited GSP‑induced depolarization. (D and G) Summary of responses to GSP. Mean ± standard error of mean. **p < 0.01. ****p < 0.0001. CTRL, control; GSP, Grape seed powder.

To determine if the PKC or PKA pathway is required for GSP‑induced ICC depolarization, we used various PKC or PKA pathway antagonists: a broad-spectrum PKC inhibitor (staurosporine), a calcium-dependent PKC α/β inhibitor (Go6976), a calcium-independent PKC δ inhibitor (rottlerin), an inhibitor of adenylyl cyclase (SQ22536), and myristoylated PKA inhibitor. In the presence of PKC inhibitors, GSP‑induced ICC depolarization was inhibited (Fig. 4A-4C). The degrees of depolarization were 17.1 ± 0.7 mV (*p* < 0.0001) by staurosporine, 15.9 ± 0.8 mV (*p* < 0.0001) by Go6976, and 22.3 ± 0.8 mV (p < 0.01) by rottlerin (Fig. 4D). In addition, in the presence of PKA inhibitors, GSP‑induced ICC depolarization was also inhibited (Fig. 4E and 4F). The degrees of depolarization were 0.7 ± 0.4 mV (*p* < 0.0001) by SQ22536, and 0.6 ± 0.5 mV (*p* < 0.0001) by myristoylated PKA inhibitor (Fig. 4G). These results showed that PKC or PKA pathway influenced GSP‑induced ICC depolarization.

### MAPK pathway antagonists influence GSP‑induced ICC depolarization

**Figure 5:**
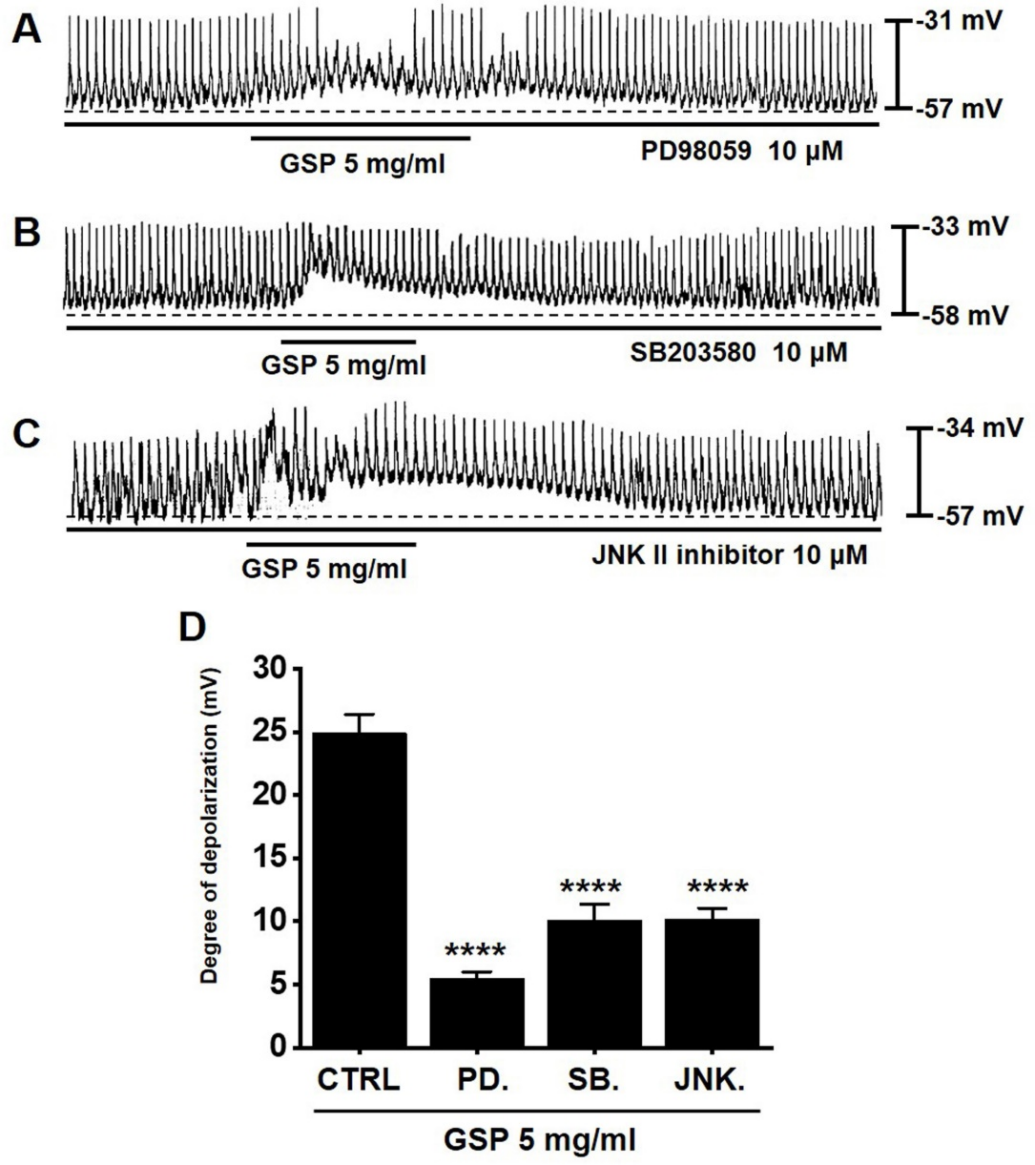
Effects of mitogen-activated protein kinase inhibitors on GSP‑induced depolarization. (A-C) PD98059, SB203580, and JNK II inhibitor blocked GSP‑induced depolarization. (D) Summary of responses to GSP. Mean ± standard error of mean. ****p < 0.0001. CTRL, control; GSP, Grape seed powder.

To determine if the MAPK pathway is required for GSP‑induced ICC depolarization, we used various MAPK pathway antagonists: a p42/44 inhibitor (PD98059), a p38 inhibitor (SB203580), and c-jun NH_2_-terminal kinase (JNK) II inhibitor. In the presence of MAPK pathway inhibitors, GSP‑induced ICC depolarization was inhibited (Fig. 5A-5C). The degrees of depolarization were 5.5 ± 0.5 mV (*p* < 0.0001) by PD98059, 10.1 ± 1.2 mV (*p* < 0.0001) by SB203580, and 10.2 ± 0.9 mV (*p* < 0.0001) by JNK II inhibitor (Fig. 5D). The results showed that the MAPK pathway influenced GSP‑induced ICC depolarization.

### GSP increases ITR in normal and GMD mice

**Figure 6:**
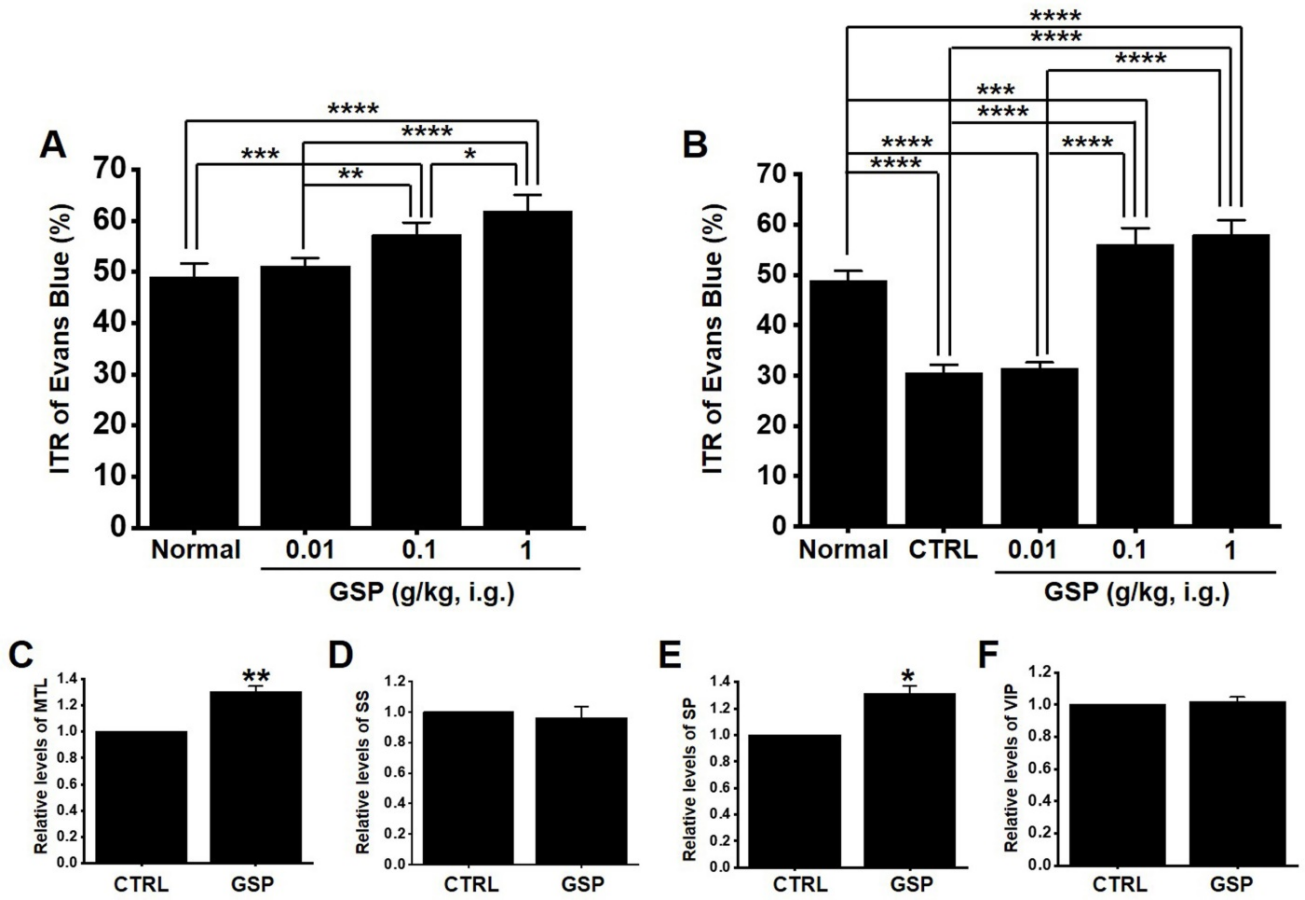
Effects of GSP on ITR and the levels of intestinal hormones in normal and GMD mice. (A) GSP increased ITR in normal mice. (B) GSP increased ITR in GMD mice. GI hormone levels of (C) MTL, (D) SS, (E) SP, and (F) VIP were measured using a radioimmunoassay. Mean ± standard error of mean. *p < 0.05. **p < 0.01. **p < 0.01. CTRL, control; GSP, Grape seed powder; ITR, intestinal transit rate; MTL, motilin; SS, somatostatin; SP, substance P; SS, somatostatin; VIP, vasoactive intestinal peptide.

In normal mice, the ITR was 50.8 ± 2.8 % (Fig. 6A), which increased with GSP treatment (51.2 ± 2.9 % at 0.01 g/kg, 58.8 ± 2.7 % at 0.1 g/kg, and 63.8 ± 3.6 % at 1 g/kg) (Fig. 5A). In addition, ITR decreased in AA-induced GMD model (29.7 ± 3.0 % *vs.* 51.2 ± 3.8 % in normal) (Fig. 6B). However, GSP restored this response to 33.1 ± 3.6 % at 0.01 g/kg, 43.7 ± 3.0 % at 0.01 g/kg, and 54.5 ± 3.3 % at 0.01 g/kg (Fig. 6B). GI hormone levels in serum were evaluated by radioimmunoassay. The levels of MTL (*p* < 0.01; Fig. 6C) and SP (*p* < 0.05; Fig. 6E) in the GI was significantly elevated, but the levels of SS (Fig. 6D) and VIP (Fig. 6F) showed no significant changes after GSP administration. These results showed that GSP-induced increase in ITR was mediated by an increase in MTL and SP in normal and GMD mice.

### Effects of GSP on the protein expression of TRPM7, TMEM16A, and c‑kit

**Figure 7:**
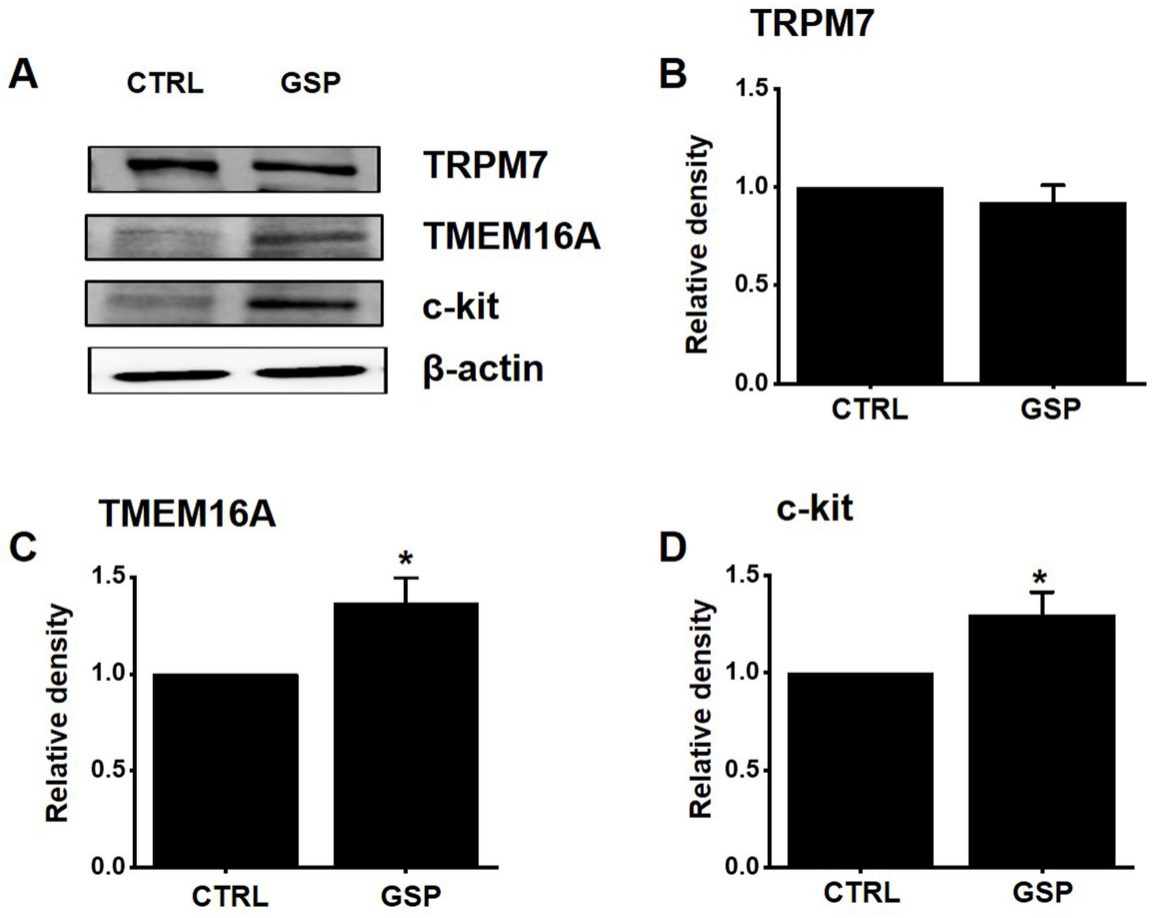
Effects of GSP on the expression of TRPM7, TMEM16A, and c‑kit. (A) Western blot results showed that the expression of TRPM7 did not change, whereas the expression of c‑kit and TMEM16A increased considerably. (B‑D) Band density relative to CTRL. Mean ± standard error of mean. *p < 0.05. CTRL, control; GSP, Grape seed powder.

TRPM7 12 and TMEM16A 19,20 channels and c-Kit 11 play important roles in the biological and physiological actions of ICCs. After treatment with GSP, the expression of TRPM7, TMEM16A, and c‑Kit was evaluated using western blotting (Fig. 7A). The expression of TRPM7 after SM treatment was almost unchanged (Fig. 7B). However, the expression of TMEM16A and c‑kit increased by about 36 % (*p* < 0.05) and 29 % (*p* < 0.05), respectively (Fig. 7C and 7D). These results showed that the GSP‑induced increase in ITR was associated with an increase in the expression of c-Kit and TMEM16A.

## Discussion

Grapes are one of the most consumed fruits worldwide. Grape seeds are rich in vitamins, fiber, and polyphenols, which are functional ingredients that can address various health issues by boosting natural physiological processes 1. Grape seed can be collected as a byproduct from any wine manufacturing industry and the seeds of red wine grapes are usually used in the preparation of GSP 1. GSP attenuates the intracellular formation of reactive oxygen species (ROS) and is considered a natural antioxidant and free radical scavenger 3. GSP improves inflammation and hyperglycemia associated with obesity 5 and suppresses the increase in body weight in C57BL/6J mice with high-fat diet (HFD)-induced obesity 21. In addition, it prevents inflammation by modulating the expression of cytokines, such as C-reactive protein, IL-6, and TNF-alpha 22. In addition, GSP prevents tumorigenesis and show chemo-preventive properties against various cancers 23,24. It may also be effective against the development of Alzheimer's disease and potentially other neurodegenerative disorders 25-27. GSP can also modulate the GI tract; it suppresses DSS-induced colitis in the intestine through the improvement of the intestinal barrier, reduction of oxidative stress, and modulation of inflammatory cytokines and gut microbiota, suggesting its potential applicability as an adjuvant therapy for ulcerative colitis 4,28. GSP has protective roles against inflammatory bowel disease through its ability to influence gut inflammation, the expression of tight junction proteins, and gut microbiota 7. In addition, GSP influences gut microbiota and enteroendocrine secretions in female rats 29. Although many studies have reported that GSP is effective in the treatment of many digestive tract disorders, few have reported the effect of GSP on GI motility.

ICCs are regarded as GI pacemaker cells 12,30,31. Abnormalities in ICCs have been implicated in GI motility disorders 12,30,31. Therefore, exploring the functions of ICCs is considered to have great significance in understanding GI motility. In the present study, we investigated the regulatory effect of GSP on physiological pacemaking in ICCs. We found that GSP depolarized pacemaker potentials and decreased their amplitudes in a concentration‑dependent manner (Fig. 2). Furthermore, we showed that pretreatment with Y25130 or SB269970 resulted in slight GSP‑induced effects. However, pretreatment with RS39604 did not influence GSP‑induced effects. In addition, pretreatment with Y25130 and SB269970 together completely blocked GSP-induced effects (Fig. 3). We also found that PKC, PKA, and MAPK pathways were involved in GSP‑induced ICC depolarization (Fig. 4 and 5). Additionally, GSP increased the ITR in normal mice and restored the ITR in GMD model mice (Fig. 6A and 6B). The level of MTL and SP in the GI was significantly elevated, but there were no changes in the levels of SS and VIP (Fig. 6C-6F). Moreover, the expression of TRPM7 after GSP treatment was almost unchanged. However, the expression of TMEM16A and c‑kit increased (Fig. 7). Therefore, through its ability to regulate GI motility, GSP is a potential adjuvant for the treatment of GI-related diseases.

ICCs function as key neural stimulators of GI motility 32. They act through various receptors of neurotransmitters and circulating hormones 32. 5-HT (serotonin) is localized in the enterochromaffin cells of the GI mucosa and within neurons in the enteric nervous system 33. As an enteric neurotransmitter, 5-HT affects neural modulation of gut smooth muscle function and may act through enteric nerves to influence GI functions 33. Thus, 5-HT participates in many physiological processes associated with digestion, including motility control. 5-HT receptors (mainly 5-HT3, 4 and 7 receptors) in ICCs have been relatively well studied 34-36. Serotonergic stimulation, one of the important contributors to the brain-gut connection, also modulates intracellular Ca^2+^ in ICCs and generates slow-wave potentials through 5-HT3, 4, and 7 receptors 37. In this study, compounds in GSP may have functioned as ligands for the receptors. Upon treatment with 5-HT3 inhibitor, Y25130, or 5-HT7 inhibitor, SB269970, GSP-induced response was suppressed (Fig. 3A and 3C); the administration of 5-HT4 inhibitor, RS39604, had no effect on the GSP-induced response (Fig. 3B). However, when Y25130 and SB269970 were administered together, the GSP-induced response was completely blocked (Fig. 3D). Therefore, GSP-induced reaction was possibly mediated by 5-HT3 and 5-HT7 receptors, and these receptors may be the target of physiological or pharmacological mechanisms for the development of therapeutic agents for diseases associated with GI motility. Muscarinic acetylcholine receptors are present throughout the GI tract and are involved in regulating the contraction of smooth muscles 38. Acetylcholine secreted from the autonomic nerves in the GI tract regulates the contraction of smooth muscle through muscarinic receptors 2 and 3, and it is known that 2 receptor is mainly present 39,40. It is also known that the 2nd and 3rd muscarinic receptors exist in ICC 41. In this study, the involvement of muscarinic receptors was not investigated. This is because the 5-HT receptor showed a sufficient response. It is thought that further research is needed in this area in the future.

According to previous studies, GSP responds through various protein kinase pathway mechanisms 42-45. In this study, the PKC, PKA, and MAPK pathways were involved in the GSP-induced response, and compared with MAPK and PKA response, PKC response was less involved (Fig. 4 and 5). PKA reaction was involved possibly due to the increased activity of the adenylate cyclase pathway mediated by 5-HT7 42,43. It has also been reported that MAPK is highly involved in various reactive mechanisms mediated by GSP 44,45. MAPK is an important signaling molecule that affects cellular responses in various fields such as proliferation, differentiation, migration, and apoptosis 46. It also plays an important role in various pathological reactions such as cancer, cardiac hypertrophy, and diabetes 46. MAPK plays a role in leading the proliferation and differentiation of cells in the GI tract and also plays a role as a therapeutic target for GI motility control by being involved in regulating the contractile response of GI smooth muscle cells and ICC 47. Therefore, it is thought that GSP-induced response is regulated by the protein kinase pathway in many cells, including ICCs.

ITR refers to the time it takes for ingested food to pass through the intestine. In this study, GSP increased ITR in normal and GMD mice (Fig. 6A and 6B). GSP regulates ICC pacemaking; hence, the increase in ITR is thought to be closely related to ICC activation (Fig. 2). To explore other possible mechanisms, we looked at changes in GI hormones. GI hormonal changes can alter GI motility 48,49. Changes in four representative hormones, MTL, SP, SS, and VIP, were investigated, and the results showed that GSP increased the levels of MTL and SP hormones, but the levels of SS and VIP hormones did not change (Fig. 6C-6F). Three cell surface proteins, TRPM7, c-Kit, and TMEM16A, are markers of ICC activation 11,12,19,20. In this study, the expression of TMEM16A and c-Kit increased after GSP administration, but the expression of TRPM7 did not change (Fig. 7). Therefore, it is thought that GSP-induced increase in ITR is related to the activation of ICC, increase in the levels of MTL and SP hormones, and the increase of the expression of TMEM16A and c-Kit.

In summary, this study showed that: i) GSP depolarized the pacemaker potentials in ICC; ii) Y25130, a 5-HT3 antagonist, and SB269970, a 5-HT7 antagonist, inhibited GSP‑induced responses; iii) PKC, PKA, and MAPK inhibitors inhibited GSP‑induced responses; iv) GSP increased ITR values in normal and GMD mice; v) GSP elevated the levels of MTL and SP hormones but had no effect on the levels of SS and VIP; vi) SM‑induced ITR increase was related to the increase in the protein expression of c‑kit and the TMEM16A proteins. Considering the problems of conventional drugs for GI motility control, there is an urgent need for a safe and natural therapeutic agent with few side effects and excellent therapeutic efficacy. Taken together, GSP may be useful in safely preventing or treating various symptoms caused by GI disorders.

## Figures and Tables

**Table 1:** The analysis condition of (-) Epicatechin, (-) Epicatechin gallate, Resveratrol, and Nomilin.

Time (minute)	0.1% FA/ Water (%)	0.1% FA/ Acetonitrile (%)	Flow rate (ml/minute)
0	98	2	0.40
1.0	98	2	0.40
2.0	95	5	0.40
3.0	85	15	0.40
5.0	75	25	0.40
6.0	60	40	0.40
8.0	50	50	0.40
9.0	20	80	0.40
10.0	10	90	0.40
12.0	2	98	0.40
14.0	98	2	0.40
16.0	98	2	0.40

**Table 2:** Concentrations of the GSE four marker compounds by UPLC

Compound			Content (Unit: mg/kg)
(-) Epicatechin			160.17 ± 2.52
(-) Epicatechin gallate			2.72 ± 0.26
Resveratrol			0.03 ± 0.0005
Nomilin			9.75 ± 0.36
